# Scalable Self-Sensing Mechanical Metamaterials by Conformal Coating of 3D-Printed Lattices with Nanocomposites

**DOI:** 10.3390/s26051670

**Published:** 2026-03-06

**Authors:** Dawn K. D. Veditz, Emma R. Merriman, Sofia Z. Anissian, Long Wang

**Affiliations:** 1 Department of Mechanical Engineering, California Polytechnic State University, 1 Grand Ave, San Luis Obispo, CA 93407, USA; 2 Department of Biomedical Engineering, California Polytechnic State University, 1 Grand Ave, San Luis Obispo, CA 93407, USA; 3 Department of Civil and Environmental Engineering, California Polytechnic State University, 1 Grand Ave, San Luis Obispo, CA 93407, USA

**Keywords:** auxetic, carbon nanotube, metamaterial, nanocomposite, piezoresistivity, 3D printing, additive manufacturing

## Abstract

**Highlights:**

**What are the main findings?**

- An efficient fabrication process conformally integrates CNT–SEBS nanocomposites onto 3D-printed flexible lattices, yielding stable and repeatable piezoresistive response under 0–40% quasi-static cyclic compression.
- Lattice topology strongly governs the strain sensing performance, where the auxetic unit cell achieved the highest sensitivity, and scaling to 8-cell auxetic lattices retained the high sensitivity.

**What are the implications of the main findings?**

- Strain sensing metamaterials can be scaled from unit cells to larger lattices while maintaining repeatable electromechanical readout, supporting large-scale and conformal sensing applications.
- A desired strain sensitivity could be engineered by tailoring the cell type using the same coating formulation and fabrication process, enabling design-driven tuning without reformulating the nanocomposites.

**Abstract:**

Metamaterials possess unique and desirable multiphysical behaviors derived from deliberately arranging conventional materials into designed structural topologies. Multifunctional mechanical metamaterials that can both carry load and provide in situ state awareness are increasingly needed for applications such as structural health monitoring and soft robotic systems. To address the demand for multifunctional metamaterials, this study reports a scalable fabrication strategy for self-sensing lattice metamaterials by conformally dip-coating 3D-printed flexible cells with a carbon nanotube (CNT)–styrene–ethylene–butylene–styrene (SEBS) nanocomposite. Scanning electron microscopy shows that the coating conforms closely to the printed struts with well-dispersed CNT networks. The electromechanical behavior of coated Octet, Kelvin, and auxetic unit cells was characterized under quasi-static cyclic uniaxial compression (0–40% strain). All the coated structures exhibited highly stable, reversible, and repeatable piezoresistive response, with a near-linear relationship between resistance change and strain. Among the tested geometries, the auxetic unit cell achieved the highest strain sensitivity that was approximately four times that of the Octet cell and six times that of the Kelvin cell. To evaluate scalability, auxetic lattices containing eight scaled auxetic unit cells were shown to retain high sensitivity and remained statistically similar to the unit cell. This study demonstrates that the strain sensing performance of nanocomposites can be engineered through lattice topology using a simple dip-coating functionalization approach, enabling scalable self-sensing metamaterials for large-scale and conformal sensing applications.

## 1. Introduction

Metamaterials have been extensively studied mainly due to their unique effective properties governed more by structural topology than by constituent composition. By deliberately arranging conventional materials into designed geometries, metamaterials can exhibit unusual thermal, mechanical, electrical, acoustic, and electromagnetic behaviors that are difficult to achieve with conventional homogeneous materials alone [1,2]. Recent advancements in additive manufacturing, particularly three-dimensional (3D) printing, have enabled the fabrication of complex lattice structures. As a consequence, metamaterials have been explored in a myriad of applications, such as thermal management, wave manipulation, energy absorption, and flexible strain sensors, and have broad impacts on biomedical applications [3,4,5]. Despite these advancements, metamaterials are primarily designed for a single function for a certain target response. However, applications such as structural health monitoring, soft robotics, and aeronautical structures are demanding multifunctional metamaterials that can both bear load and provide state awareness (e.g., deformation or damage) [6,7,8]. In particular, metamaterials that deliberately couple mechanical deformation to a measurable electrical signal remain relatively underdeveloped. Towards addressing this gap, He et al. created high-stiffness self-sensing lattice structures utilizing metal plated-vat photopolymerization 3D printing [9]. They reported that the self-sensing lattices experienced maximum loads on the order of 1 kN, but experienced plastic behavior beyond 5% strain, and thus sensing results were limited to 0–2% strain [9]. While metal plating was promising, this functionalization strategy could compromise the flexibility of these 3D structures and limit their applications.

Another promising approach to achieving multifunctional electromechanical behavior is the incorporation of flexible conductive nanomaterials. Nanomaterials, such as carbon nanotubes (CNTs), can establish percolated conductive networks within polymeric matrices, forming piezoresistive nanocomposites whose electrical resistance varies with deformation [10,11,12,13]. These nanocomposites are attractive for strain sensing because they can easily form continuous sensing layers and remain mechanically compliant as compared to traditional discrete strain gauges. For instance, Zhao et al. investigated flexible self-sensing piezoresistive sensors using multi-walled CNTs capable of measuring strain up to 60%, but could not function as a 3D structural element [14]. In fact, current nanocomposites are typically cast or printed into planar films, which can limit the achievable deformation modes and constrain sensor form factors. In addition, to engineer the piezoresistive performance of nanocomposites, current methods still heavily rely on trial and error. However, recent developments in intelligent metamaterials highlight the importance of clearly defined architecture–property relationships to support future computational designs [15].

In the authors’ prior work [16], the sensing performance of piezoresistive nanocomposites was successfully tuned over a wide range through a topological design framework without changing the underlying material system. It was discovered that structural geometry can be treated as a controllable design variable for tailoring bulk piezoresistive response, complementing (and potentially reducing reliance on) empirical, composition-driven approaches. This insight motivates this study to extend “topological tuning” from planar nanocomposite films to 3D architected structures.

Cellular lattice structures are of particular interest in this study, as their unit cell topology governs deformation modes, stress/strain localization, and load path in ways that are not accessible in 2D layouts. Conventional structures, including Kelvin and Octet Truss cells, generally exhibit positive Poisson’s ratio behavior, with lateral expansion under compression and contraction under tension [17]. However, auxetic cells are designed to achieve negative Poisson’s ratio, resulting in a reduction in cell width under uniaxial compression or an expansion of cell width when subjected to uniaxial tension [18,19]. This auxetic deformation behavior has been shown to enhance strain sensing capabilities [20]. Nevertheless, studies on the systematic integration of piezoresistive nanocomposites with 3D lattice architectures remains relatively limited, especially for achieving scalable fabrication that preserves both mechanical compliance and stable sensing behavior. In addition, there is a knowledge gap about how lattice topologies (including auxetic versus non-auxetic cells) influence electromechanical sensitivity when the same coating material system and processing conditions are held constant.

Therefore, the objective of this study is to develop and validate a scalable approach for fabricating self-sensing mechanical metamaterials by functionalizing 3D-printed lattices with a CNT-based nanocomposite coating, and to quantify how lattice geometry governs electromechanical sensing behavior and experimentally determine if past results showing increased sensitivity in auxetic structures continue to apply in multidimensional lattice structures. To be specific, flexible structures are fabricated via 3D printing and dip-coated with a CNT-based nanocomposite to form conductive, deformable sensing coating. The resulting coated structures are then characterized mechanically and electromechanically under cyclic compressive loading. Finally, to assess geometric and functionalization scalability, auxetic lattices composed of multiple scaled unit cells are fabricated and evaluated to determine whether high strain sensitivity can be retained at the lattice level. Overall, this study shows promise in establishing a practical framework for engineering multifunctional, self-sensing metamaterials, where sensing performance can be tuned primarily through mechanical architecture.

## 2. Materials and Methods

### 2.1. Materials

The MWCNTs used in this study were obtained from Cheap Tubes, Townshend, VT, USA, (outer diameter: <8 nm; length: 10–30 μm; and purity: >98%); toluene and styrene–ethylene–butylene–styrene (SEBS) were from Sigma-Aldrich, Burlington, MA, USA; disposable laboratory supplies were obtained from Fisher Scientific, Pittsburgh, PA, USA. The electrodes were constructed using conductive thread from AdaFruit, Brooklyn, NY, USA, and conductive silver paint from Ted Pella, Redding, CA, USA.

### 2.2. Cell Fabrication

**Figure 1:**
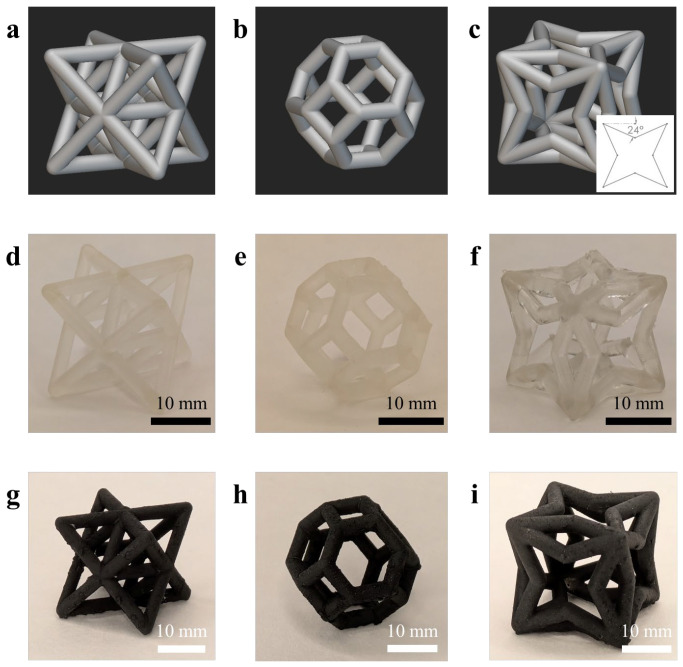
The nTop designs of (**a**) Octet, (**b**) Kelvin, and (**c**) auxetic unit cells. The inset figure in (**c**) shows the strut angle within the auxetic cell. Photos of 3D-printed (**d**) Octet, (**e**) Kelvin, and (**f**) auxetic unit cells. Photos of nanocomposite-coated (**g**) Octet, (**h**) Kelvin, and (**i**) auxetic unit cells.

The lattice cells were first designed using version 5.34.3 of the nTop software, where each cell model was generated as a mesh file. nTop includes a default unit cell pattern for the Octet and Kelvin cells; nTop’s default unit cell patterns were used, as shown in Figure 1a,b. However, the cell pattern of the 3D auxetic cell used in this study was modeled manually, as shown in Figure 1c. In particular, the angle of the struts bend toward the middle of the cell face was selected to be 24° to ensure that auxetic behavior was established. All cells had overall dimensions of 20 × 20 × 20 mm^3^ and a strut diameter of 3 mm. The cells were meshed with a tolerance of 0.05 mm and a minimum feature size of 0.05 mm and then exported for 3D printing.

**Figure 2:**
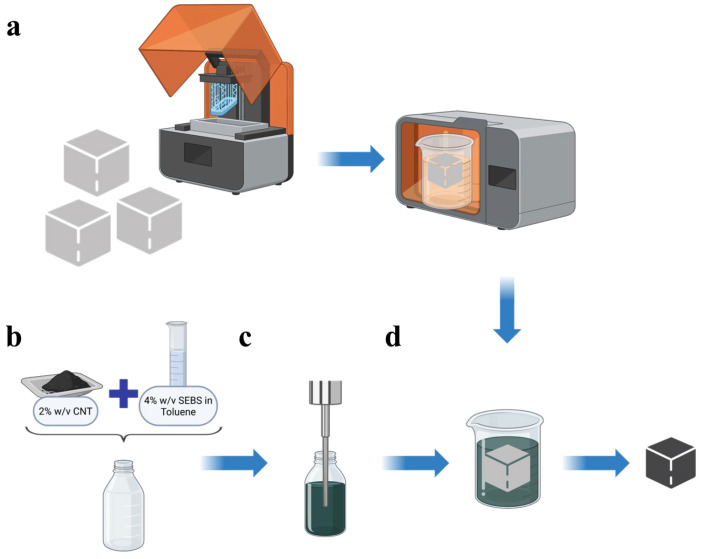
Schematics of the fabrication process. (**a**) 3D printing and post-processing of the lattice structures. (**b**) Mixing CNTs with the SEBS/toluene solution. (**c**) Ultrasonication of the CNT–SEBS/toluene mixture for dispersing the CNTs. (**d**) Dipping coating the 3D lattice structures with the CNT-based solution, forming coated samples.

Figure 2 illustrates an overview of the fabrication procedures developed in this study (schematics designed using the BioRender.com webservice). To be specific, the designed structures were printed with Formlabs, Union City, CA, USA, Form 4 3D printer using the as-purchased Elastic 50A Resin (Figure 2a). The print layer thickness was 100 µm to ensure high fabrication accuracy. Each structure was printed in an orientation of ~30 degrees about its axis to minimize unsupported minima (i.e., localized low regions or hanging regions of the model), which provided consistent printing quality [21]. Figure 1d–f exhibit the post-processed and fully cured unit cells. One can observe that the printing process delivered high printing fidelity compared to the designed models.

### 2.3. Nanocomposite Coating Fabrication

The nanocomposite coating ink was prepared by dispersing MWCNTs in an organic solution. First, SEBS was dissolved in toluene solvent at a concentration of 4% *w*/*v* via mechanical stirring for 24 h at room temperature using a magnetic stir bar from Fisher Scientific (Figure 2b). Then, the MWCNTs were dispersed into the solution by 30 min of ultrasonication to achieve a 2% *w*/*v* concentration of CNT–SEBS/toluene ink (Figure 2c).

The cured 3D structures were then coated with the nanocomposite ink via dip-coating (Figure 2d). It was found that dip-coating twice with a 15 min drying in between could provide the most consistent coating quality and optimal electromechanical performance. All the coated samples were air dried for at least 2 h to fully evaporate the toluene solvent. Figure 1g–i show example coated unit cells. The microstructure of the nanocomposite coating was inspected using scanning electron microscopy (SEM) from Thermo Fisher Scientific, MA. USA. In addition, to prepare for strain sensing tests, conductive threads were affixed to the opposing corners of each dried sample using conductive silver paint to ensure physical and electrical contact was maintained.

### 2.4. Mechanical and Electromechanical Characterization

To investigate the effects of structural design on the mechanical and electromechanical behavior of nanocomposite-coated 3D structures, this study first subjected each unit cell to cyclic compression tests with compressive strain ranging from 0% and 40% using a Shimadzu EZ-LX universal tensile machine, from OR. USA, with a transducer of 5 kN load capacity that had ±0.5% of force measurement error. Each sample (including uncoated and coated ones) was initially preloaded with 1 N to ensure contact with the compression plates and was then deformed with a strain rate of 2%/s. The resulting force and compression displacement were recorded using TrapeziumX software version 1.5.6., which were used to analyze the samples’ mechanical properties.

**Figure 3:**
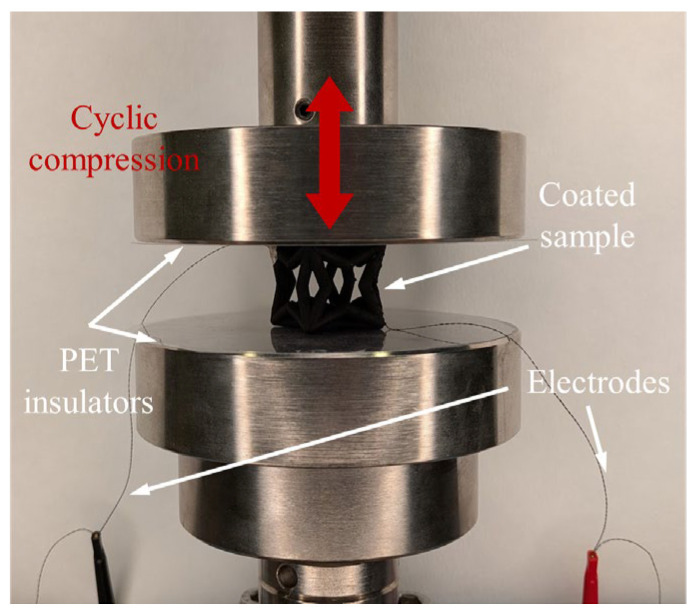
A photo of the electromechanical sensing experiment test setup with a nanocomposite-coated auxetic unit cell mounted between the compression plates, electrically isolated by two non-conductive polyethylene terephthalate (PET) sheets.

In addition, for the nanocomposite-coated samples, a Keysight 34465A Digital Multimeter, from CA. USA, was connected to each sample’s electrodes to record the two-probe resistance time-series data during the compression cycles. This electromechanical test setup is depicted in Figure 3. Note that the first 10 loading cycles were excluded from analysis to eliminate the initial transient behavior of the material. Furthermore, affixing the electrodes to opposing corners was done to encourage a consistent and comparable overall current flow through the sample. While localized current distribution may vary with topology, all samples were tested under identical electrical and mechanical conditions to enable comparison of topology-dependent electromechanical responses.

### 2.5. Statistical Analysis

To rigorously compare the electromechanical sensing results across different mechanical designs, version 18.2.0 of the JMP software was used to perform analysis of variance (ANOVA) with post hoc Tukey analysis on the calculated sensitivity values of each structure. Four samples per each of the four structure types were tested, *n* = 4 and *k* = 4, and the value of *p* < 0.05 was used to determine statistically significant differences. A power analysis for the ANOVA with N = 16 was then also used to ensure the ANOVA could adequately determine statistically significant differences.

## 3. Results and Discussion

### 3.1. Microstrucure of the Nanocomposite Coating

**Figure 4:**
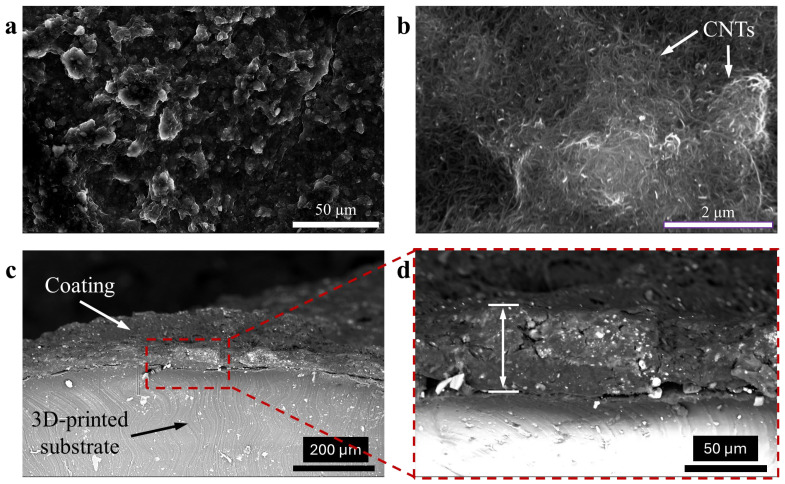
SEM images of (**a**,**b**) top and (**c**,**d**) cross-sectional views of the nanocomposite coating on a 3D-printed strut.

Figure 4a,b are the SEM images of the surface of a coated strut, which show that the CNTs were dispersed well in the nanocomposite matrix. The larger spherical features are consistent with other reported SEM images of SEBS-based composites [22,23]. The cross-sectional views of a coated strut are shown in Figure 4c,d. It can be seen that the CNT–SEBS nanocomposite coating was ~50 µm thick and conformed closely to the curved surface of the 3D-printed cylindrical strut. Overall, the SEM images indicated that the fabrication technique developed in this study successfully integrated the 3D-printed lattice structure with nanocomposite coating, forming promising multifunctional metamaterials.

### 3.2. Mechanical Behavior

**Figure 5:**
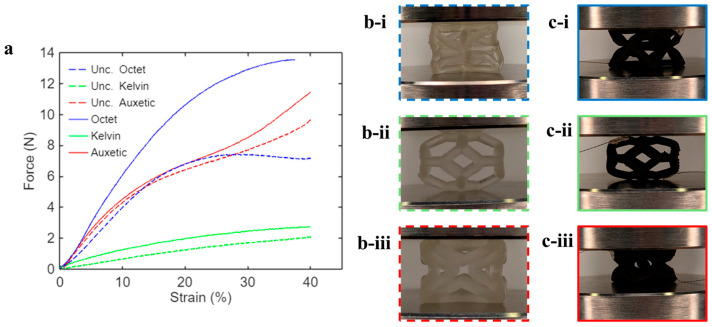
(**a**) Representative force–strain curves of uncoated (dashed lines) and coated (solid lines) unit cells. Photos of uncoated (**b-i**) Octet, (**b-ii**) Kelvin, and (**b-iii**) auxetic cells and nanocomposite-coated (**c-i**) Octet, (**c-ii**) Kelvin, and (**c-iii**) auxetic cells under 30% compressive strain.

Figure 5a shows the representative force–strain curves of uncoated and coated unit cells. The auxetic and Kelvin cells became slightly stiffer after the coating was applied, but the overall mechanical responses maintained their intrinsic features. However, Octet cells became significantly stiffer after coating, which may be due to the buckling of their struts under large compressive deformation. For instance, under 30% compressive strain, Octet cell struts buckled and rotated about their connections (Figure 5(b-i,c-i)). The nanocomposite coating was hypothesized to perform as a stretchable “skin” that could constrain the buckling and rotation of the struts, resulting in higher mechanical resistance in the coated Octet cells. On the other hand, the Kelvin and auxetic cells simply bulged outward or collapsed inward, respectively (Figure 5(b-ii,b-iii,c-ii,c-iii)), without significant strut buckling or rotation. The mechanical reinforcing effect of the nanocomposite coating was not as significant in the Kelvin and auxetic cells. Regardless of the structures, the nanocomposite coating could deform compliantly with the 3D substrates without visible cracking or delamination.

### 3.3. Electromechanical Response

To investigate the electromechanical sensing response of the coated 3D structures to uniaxial strain, resistance time histories as well as compression and force time histories were recorded simultaneously and processed. The difference in strain sensitivity between cells was investigated based on the change in the normalized change in resistance with respect to strain.

**Figure 6:**
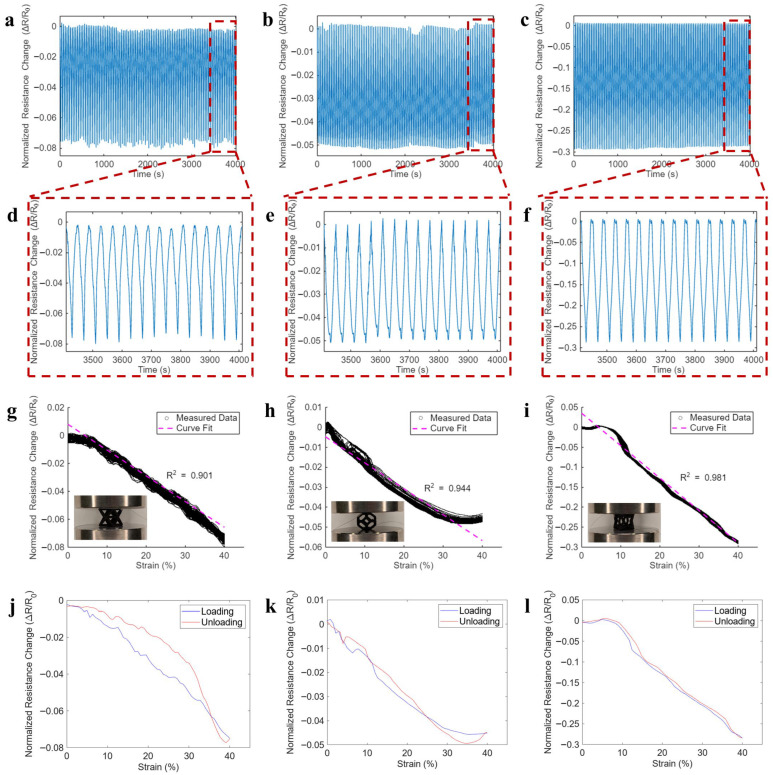
Representative time histories of normalized resistance changes of (**a**) Octet, (**b**) Kelvin, and (**c**) auxetic cells subjected to 100 cycles of compression. (**d**–**f**) Zoomed-in views of 15 loading cycles sensing response corresponding to (**a**–**c**), respectively. (**g**–**i**) Extracted normalized resistance change data from the 100 loading processes in (**a**–**c**), respectively, overlapped with least-square linear fits (dashed lines). (**j**–**l**) Representative normalized resistance changes during one cycle of loading and unloading processes to show potential hysteresis in the sensing response of all cell types.

To be specific, the normalized change in resistance (Δ*R_n_*) was first calculated using the following:

(1) $$∆R_{n}=\frac{R_{i}-R_{0}}{R_{0}}$$

where $R_{0}$ is the resistance of the unit cell at the beginning of the compression cycle at zero strain and $R_{i}$ is the resistance measured during the loading process. Figure 6a–c show the representative Δ*R_n_* time histories of Octet, Kelvin, and auxetic unit cells subjected to 100 loading cycles, respectively. Figure 6d–f are zoomed-in views of the last 15 loading cycles corresponding to Figure 6a–c, respectively. Additionally, as shown in Figure 6j–l, it is notable that, except for the Octet cell, there was very little visible hysteresis between the sensing responses to the loading and unloading processes. In the case of the Octet cell, the limited hysteresis may be due to the combination of strut buckling and bending under large deformations, causing additional localized strain, as seen in Figure 5(b-i,c-i). While this was tested under quasi-static loading conditions, this low-hysteresis sensing response could be valuable in sensing applications where instantaneous measurements are desirable. Regardless of the cell structures, all the coated samples exhibited highly stable, reversible, and repeatable electromechanical sensing response. The resistance would decrease under compression and increase back to its original state upon unloading.

In addition, the strain sensitivity (*S*) can then be defined via the following equation:

(2) $$S=\frac{∆R_{n}}{\varepsilon }$$

where *ε* is the strain measured for each value of $\Delta R_{n}$. To calculate the value of *S* for each cell, a linear least-squares regression was fitted to the $\Delta R_{n}$ data to find an approximated linear relationship between ${\Delta R}_{n}$ and ε across all the loading cycles. Figure 6g–i plot the Δ*R_n_* data extracted from 100 loading processes for Octet, Kelvin, and auxetic unit cells as a function of the compressive strains, respectively. The tight distribution of the Δ*R_n_* data points, especially those of the auxetic cells, further confirm the achieved electromechanical response was remarkably repeatable with minimal drifts. The high *R*^2^ values of linear fit (all above 0.9) indicate that it is reasonable to approximate the strain sensing response as linear.

**Figure 7:**
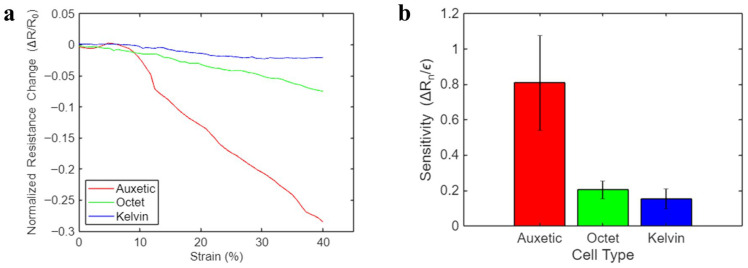
(**a**) Comparison of representative sensing responses of all the cell types to a single loading process. (**b**) Calculated mean strain sensitivities and their standard deviations of all the cell types.

To better visualize the difference in strain sensitivity of different cells, Figure 7a displays the Δ*R_n_* data of all the cell types from one loading process. According to Equation (2), the slope of each linear fit represents the sensitivity of the sample. Thus, the steeper the Δ*R_n_ − ε* curve is, the higher strain sensitivity the cell type processes. Figure 7b shows the calculated mean *S* and the standard deviations of all the cell structures across 100 loading cycles, and the corresponding numerical values are summarized in Table 1. It is clear that the auxetic unit cell possessed a significantly higher strain sensitivity, which was approximately four times that of the Octet unit cell, and six times that of the Kelvin unit cell. It is worth mentioning that the standard deviations mainly stemmed from slight variations in the samples fabricated in different batches. While mechanical metamaterials are often associated with nonlinear responses, the cell types tested in this study showed an approximately linear mechanical response within the tested strain range (Figure 5a). Nevertheless, their architecture enabled the nanocomposite-coated structures to be flexible and self-sensing under relatively large deformations. In addition, owing to the lattice architecture, these 3D sensing structures could potentially be used to bear and measure multi-directional loads. In this study, the value of the auxetic architecture was its ability to alter the electromechanical performance through structural design, as shown in Figure 7, rather than in producing strong nonlinear mechanical behavior.

Due to individual sample variation and differences in topology between cell groups, the raw resistance measurements were not directly comparable. The variations in the mean $R_{0}$ values for all cell types are summarized in Table 2. In contrast, the normalized resistance change (Δ*R_n_*) is a proportional change that isolates variations in individual cell resistance, allowing more accurate comparison. Additionally, it was determined that the total contact resistance from the electrodes was on the order of 1 Ohm, which was determined to be of negligible magnitude when normalizing the resistance change.

### 3.4. Lattice Comparison

**Figure 8:**
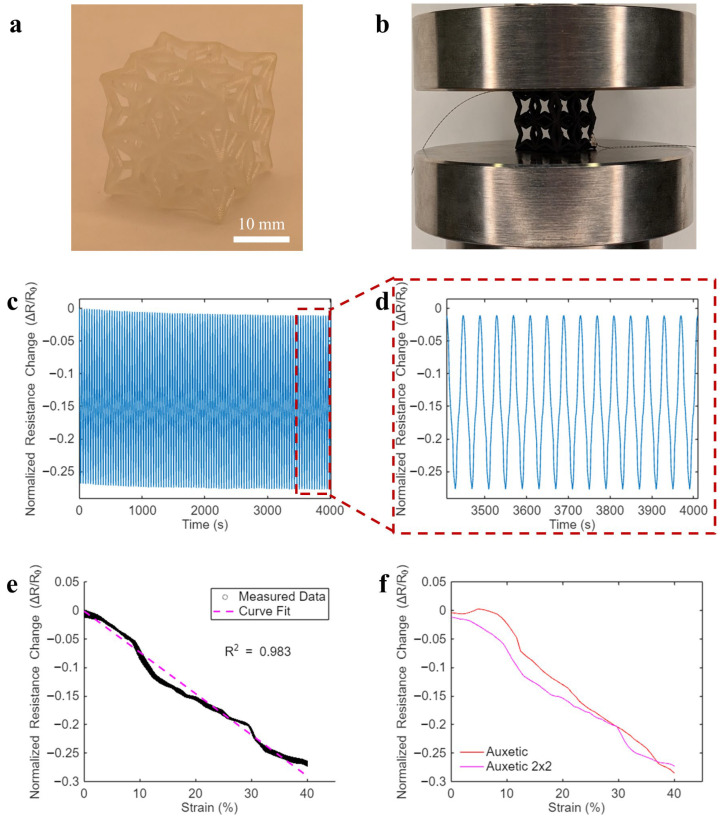
Photos of (**a**) a 3D-printed auxetic lattice and (**b**) a coated auxetic lattice mounted between the compression plate for electromechanical sensing tests. (**c**) A representative time history of normalized resistance change in an auxetic lattice subjected to 100 cycles of compression. (**d**) A zoomed-in view of sensing response to the last 15 loading cycles. (**e**) Normalized resistance change data extracted from the 100 loading process in (**c**) overlapped with a least-square linear fit. (**f**) Representative sensing responses of an auxetic unit cell and an auxetic lattice to a single compression loading process.

Since auxetic unit cells exhibited the highest strain sensitivity, this study further used this cell type to design and fabricate auxetic lattices. Here, the lattices were of the same volume (20 × 20 × 20 mm^3^) and contained eight auxetic unit cell structures. All linear dimensions of the cells in the lattice were halved from the original unit cell to maintain the same aspect ratio, resulting in strut diameters being 1.5 mm. Figure 8a shows the 3D-printed lattice, which was coated with the CNT–SEBS nanocomposite following the fabrication procedures demonstrated in Figure 2. In addition, the coated lattices were subjected to the same electromechanical tests as outlined in Section 2.3, and Figure 8b illustrates a coated lattice mounted in the test setup.

One can observe from Figure 8c,d that the auxetic lattice still exhibited highly stable and repeatable strain sensing response under 100 loading cycles for quasi-static loading conditions. In addition, Figure 8e plots the Δ*R_n_* data extracted from 100 loading processes as a function of the compressive strains, where the Δ*R_n_* data not only follows a tight distribution (i.e., high repeatability under cyclic loading) but can also be characterized as a linear relationship with compressive strain. Furthermore, to visualize the difference in strain sensitivity, Figure 8f displays the representative Δ*R_n_* data of a lattice and an auxetic unit cell from one loading process, which shows high similarity in the sensing response. Furthermore, Table 1 also includes the calculated mean sensitivity and its standard deviation of auxetic lattices. While the lattices had slightly lower sensitivity than the auxetic unit cell, they were still significantly more sensitive than Octet and Kelvin cell types. It indicates that scaling up the auxetic unit cell into lattices could effectively retain high strain sensitivity, which paves the way for future large-scale sensing applications.

### 3.5. Statistical Analysis

The results of the ANOVA with Tukey analysis are summarized in Table 3. The power analysis for the one-way ANOVA for four groups, each with *n* = 4 for a total N = 16, with α = 0.05 indicated that the study had 80% power to detect an effect with Cohen’s f = 0.97 (η^2^ ≈ 0.49). The estimated effect size for the observations was Cohen’s f ≈ 1.70 (η^2^ ≈ 0.74), meaning there was adequate power to detect an overall group effect. Table 3 also provides the lower and upper bounds of difference in sensitivity at a 95% confidence level (CL). The auxetic unit cell and auxetic lattice were found to be not statistically different, nor were the Kelvin and Octet cells. However, all of the other pairs have sensitivities that were statistically different, which is consistent with the observed difference in sensitivity (Figure 7). In other words, the major findings from the ANOVA are that (1) coated auxetic cells have a statistically significant difference in strain sensitivity compared to Kelvin and Octet cells (i.e., more sensitive) and (2) scaling up auxetic unit cells into lattices did not statistically alter their strain sensitivity. Note that the auxetic lattice was still significantly more sensitive than either the Kelvin or Octet unit types, which indicates that the base auxetic cell design retained its higher sensitivity when scaled up to the tested dimensions.

## 4. Conclusions

This study developed and implemented an efficient fabrication technique to consistently integrate CNT–SEBS nanocomposite thin films with 3D-printed structures. The nanocomposite coating conformed to the complex 3D substrate structures and could deform compliantly with the structures. This fabrication strategy enabled the functionalization of mechanical metamaterials, endowing them with electromechanical sensing behavior. These self-sensing mechanical metamaterials can open tremendously more new opportunities to develop multifunctional structures that can strategically bear and monitor deformations and loads, which can be highly valuable for civil, mechanical, aerospace, and biomedical applications.

In addition, this study demonstrates that the strain sensitivity of coated 3D structures was dependent on the design of the structures. To be specific, although all the samples were fabricated with the same materials following the same procedures, the chosen auxetic design was ~4 times more sensitive to compression than the Octet cell design, and ~6 times more sensitive than the Kelvin cell design, which are statistically different, as verified through the ANOVA. Furthermore, it was found that scaling up the auxetic unit cell into lattices could still retain its high strain sensitivity, laying the foundation for future large-scale mechanical sensing applications.

Future studies will focus on optimizing the design of mechanical metamaterials to facilitate scalable coating of nanocomposites and to achieve target mechanical and electromechanical sensing response. In addition, the lattice structures will be further scaled to include more unit cells, which then requires a more effective measurement scheme than the two-probe measurement used in this study to better extract sensing signals.

## Figures and Tables

**Table 1:** Mean sensitivity results for each cell type.

Cell Type	Mean Sensitivity ^1^	Standard Deviation ^1^
Octet	−0.206	0.049
Kelvin	−0.137	0.050
Auxetic	−0.814	0.299
Auxetic Lattice	−0.611	0.119

^1^ Units are defined by normalized resistance change divided by strain, both unitless.

**Table 2:** Mean initial resistance values for each cell type.

Cell Type	Mean R_0_ (Ω)	Standard Deviation (Ω)
Octet	337	161
Kelvin	211	83
Auxetic	346	89
Auxetic Lattice	506	103

**Table 3:** Statistical significance values calculated between each pair of structure types.

Cell Type	Cell Type	*p*-Value	Significant Difference	Lower CL Bound	Upper CL Bound
Auxetic	Kelvin	0.0004	Yes	0.326	1.028
Auxetic	Octet	0.0011	Yes	0.257	0.959
Auxetic Lattice	Kelvin	0.0115	Yes	0.123	0.825
Auxetic Lattice	Octet	0.0310	Yes	0.054	0.756
Auxetic	Auxetic Lattice	0.3662	No	−0.147	0.555
Octet	Kelvin	0.9465	No	−0.282	0.420

## Data Availability

Dataset available upon request.

## Abbreviations

The following abbreviations are used in this manuscript:

3D	Three-dimensional
ANOVA	Analysis of variance
CNT	Carbon nanotubes
SEBS	Styrene–ethylene–butylene–styrene
SEM	Scanning electron microscopy
